# Supplementary material for: Dynamic analysis of the individual patterns of intakes, voids, and bladder sensations reported in bladder diaries collected in the LURN study
Source: PLoS One. 2023 Nov 20;18(11):e0284544. doi: 10.1371/journal.pone.0284544 (PMC10659201; doi:10.1371/journal.pone.0284544)
Supplement: S1 Appendix — (PDF) [file pone.0284544.s003.pdf]

Project A: Voiding/Intake Diary

A: Project A: Voiding/Intake Diary		
1	Did the participant complete a voiding and intake diary?	O Yes, completed paper diary<br>O Yes, completed Vesica diary<br>O No → **Go to B1**
2	If yes, please enter the following information from the voiding/intake diary:	

Date of entry:	Time of entry:	Is this an entry in which the participant woke up?	Is this an entry in which the participant went to bed?	Drink amount :	Drink type:	Urine output:	Bladder sensation:	Leak (stress, urge, or unknown/other)	Is this an entry in which the participant changed pads?
		O No<br>O Yes	O No<br>O Yes						O No<br>O Yes
		O No<br>O Yes	O No<br>O Yes						O No<br>O Yes
		O No<br>O Yes	O No<br>O Yes						O No<br>O Yes
		O No<br>O Yes	O No<br>O Yes						O No<br>O Yes
		O No<br>O Yes	O No<br>O Yes						O No<br>O Yes

3	When completing the diary, did the participant avoid caffeine due to their lower urinary tract symptoms (LUTS)?	O No<br>O Yes<br>O Unknown
4	When completing the diary, did the participant avoid alcohol due to their LUTS?	O No<br>O Yes<br>O Unknown

B: Questionnaire Complete		
1	Questionnaire Complete	O Yes
